# A simplified method for preventing postmortem alterations of brain prostanoids for true *in situ* level quantification

**DOI:** 10.1016/j.jlr.2024.100583

**Published:** 2024-06-21

**Authors:** Derek Besch, Drew R. Seeger, Brennon Schofield, Svetlana A. Golovko, Meredith Parmer, Mikhail Y. Golovko

**Affiliations:** Department of Biomedical Sciences, University of North Dakota, Grand Forks, ND, USA

**Keywords:** arachidonic acid, brain Lipids, cyclooxygenase, lipidomics, prostaglandins

## Abstract

Dramatic postmortem prostanoid (PG) enzymatic synthesis in the brain causes a significant artifact during PG analysis. Thus, enzyme deactivation is required for an accurate *in situ* endogenous PG quantification. To date, the only method for preventing postmortem brain PG increase with tissue structure preservation is fixation by head-focused microwave irradiation (MW), which is considered the gold standard method, allowing for rapid in situ heat-denaturation of enzymes. However, MW requires costly equipment that suffers in reproducibility, causing tissue loss and metabolite degradation if overheated. Our recent study indicates that PGs are not synthesized in the ischemic brain unless metabolically active tissue is exposed to atmospheric O_2_. Based on this finding, we proposed a simple and reproducible alternative method to prevent postmortem PG increase by slow enzyme denaturation before craniotomy. To test this approach, mice were decapitated directly into boiling saline. Brain temperature reached 100°C after ∼140 s during boiling, though 3 min boiling was required to completely prevent postmortem PG synthesis, but not free arachidonic acid release. To validate this fixation method, brain basal and lipopolysaccharide (LPS)-induced PG were analyzed in unfixed, MW, and boiled tissues. Basal and LPS-induced PG levels were not different between MW and boiled brains. However, unfixed tissue showed a significant postmortem increase in PG at basal conditions, with lesser differences upon LPS treatment compared to fixed tissue. These data indicate for the first time that boiling effectively prevents postmortem PG alterations, allowing for a reproducible, inexpensive, and conventionally accessible tissue fixation method for PG analysis.

Accurate quantification of prostanoids (PG), including prostaglandins, prostacyclins, and thromboxanes, is important because they regulate many physiologically and pathologically relevant processes such as inflammation, pain, fever, sleep, neurogenesis, angiogenesis, and cerebral blood flow (1, 2, 3, 4). However, a major artifact in quantifying PG is their dramatic postmortem increase (5, 6, 7). This postmortem increase in PG was traditionally thought to occur *in situ* before craniotomy as a result of ischemia (8, 9). Thus, it was assumed that rapid deactivation of cyclooxygenase (COX) *in situ* using freeze-blowing with liquid N_2_ or heat denaturation with focused microwave irradiation (MW) is required to prevent postmortem PG formation (10).

Although effective, freeze-blowing is a technically challenging procedure and disrupts brain morphology, precluding regional analysis. In addition, frozen tissue retains the potential for postmortem induction of PG, as enzymes can become active during thawing and further sample handling (7, 11). Alternatively, fixation with MW effectively prevents further metabolic activity during sample handling and preserves brain morphology if the tissue is not overheated. For this reason, MW has become the gold standard in tissue fixation for the study of PG by rapidly (<1.5 s) increasing temperatures to 90°C (5, 7).

However, during the last two decades of utilizing MW (3, 7, 11, 12, 13, 14, 15, 16), we have noticed considerable limitations, including high variability in MW outcomes. Excessive and rapid heating often causes tissue loss by expelling brain matter out of the skull into the holding chamber, preventing regional metabolic analysis and potentially causing metabolome alterations. Several factors that are difficult to control may contribute to this variability, including high energy demand which may not always be adequately supplied, inconsistent MW recovery after each use, and the deleterious effect of small differences in animal size and position in the MW chamber. In addition, MW equipment is expensive and hard to acquire due to the limited market. This problematic combination necessitated the development of a novel fixation method that is easily reproducible, reliably preserves brain tissue architecture, inexpensive, and accessible to virtually any lab.

Our recent study demonstrated that despite a common misconception, PGs are not induced *in situ* during global brain ischemia but rather are increased as a result of exposing metabolically active tissue to exogenous O_2_ during tissue removal (16). Therefore, we hypothesized in the present study that it is not necessary to rapidly deactivate enzymes after decapitation, provided the tissue is not exposed to atmospheric O_2_. Based on this hypothesis, we proposed that boiling tissue to heat denature enzymes *in situ* could be used as an alternative method to fix tissue for PG analysis. To test this method, we compared basal and LPS-induced PG levels in boiled, MW, and unfixed tissue. We report that 3 min boiling completely prevented postmortem brain PG synthesis. Basal PG levels were not different between boiled and MW brain tissues. After LPS treatment, PGE_2_, PGD_2_, PGF_2α_, and 6keto-F_1α_ levels were similarly and significantly increased in boiled and MW brains when compared to basal levels. However, in unfixed tissues, basal levels were significantly increased due to postmortem synthesis that obscured the effect of LPS on PGF_2α_ and 6keto-F_1α_, with lesser changes in PGE_2_ compared to tissue fixed by boiling or MW. These results indicate that boiling is an effective method for tissue fixation before PG quantification, and has additional benefits of being inexpensive, accessible, and reproducible. Further studies are required to validate the efficacy of this method in preventing postmortem alterations in other labile metabolites.

## Materials and methods

### Animals

The handling and treatment of mice in this study were conducted in accordance with the National Institutes of Health Guidelines for the Care and Use of Laboratory Animals and a protocol approved by the University of North Dakota IACUC (protocol 2103-5). Twenty-two male and 20 female (6–8 month old) C57BL/6 mice were randomly assigned to experimental groups. Our unpublished data indicate no differences in the basal or induced PG levels between male and female mice. Mice were provided with standard laboratory chow and water ad libitum.

### Temperature recording in the brains placed in boiling saline

To determine the dynamics of temperature in the brain after decapitation into boiling saline, a silicone-insulated wire thermocouple (Control Company) was inserted into the deep brain after decapitation, tied in place with surgical thread, and then incubated between two heating pads (Gaymar/Stryker) maintained at 37°C for brain internal temperature equilibration, reflecting near-physiological temperatures at the moment of decapitation (17). Probed mouse brains were placed into 500 ml of boiling saline. The temperature was recorded every 15 s for a total of 5 min. Afterward, brains were inspected to confirm the placement of the probe within the center of the brain. Saline was used to maintain iso-osmolarity during boiling, preventing alterations to metabolite concentrations caused by osmosis.

### PG synthesis activity assay after fixation with boiling

Mice were decapitated directly into boiling saline and heads were boiled for a total time of 1.5 or 3 min or submerged in a beaker of saline equilibrated in a water bath held at 80°C for 3 or 6 min. After boiling or heating to 80°C, heads were placed on ice for ∼5 min, the whole brains were removed, frozen in liquid N_2_, and pulverized to a powder under liquid N_2_. To check PG synthesis activity after boiling, samples of pulverized brain tissue (∼20 mg) were homogenized in a Tenbroeck homogenizer containing 1 ml of saline with 1 ng of PGE_2_-d_9_ (Cayman Chemical Co) and incubated in a water bath at 37°C for 20 min. Individual PG were quantified using the ultra-high-pressure liquid chromatography-mass spectrometry (UPLC-MS) method described below. In other experiments, 3 min boiling was used.

### Animal treatment with LPS and brain tissue fixation

Animals were administered 3.3 μl/g body weight (gbw) saline or 3.3 μl/gbw LPS in saline (0.3 mg/ml, *Escherichia coli* O55:B5, product number L2880, Sigma, St. Louis, MO) via intraperitoneal (i.p.) injection and monitored for signs of a febrile response (isolation, shaking, limited movement) (18, 19) for 3 h to induce brain PG synthesis (20, 21, 22, 23). Animals were then anesthetized by isoflurane and either exposed to MW (2.8 kW, 1.5 s; 6 kW Microwave Generator S6GB/11404, Cober Electronics, Inc) or decapitated directly into boiling saline and boiled for 3 min to denature enzymes in situ. Another set of mice was anesthetized by isoflurane and euthanized by decapitation without MW or boiling (unfixed group). For each experimental condition, brains were removed following craniotomy, exposed to room air for 30 s, frozen in liquid phase N_2_, and processed as described above.

### PG analysis

Brain PGs were extracted and quantified as previously described (16). Briefly, pulverized tissue (∼20 mg) was homogenized in 3 ml of 2:1 acetone:saline with 1 ng of PGE_2_-d_9_ as an internal standard. For the analysis of PG induction during tissue incubation, pulverized tissue (∼20 mg) was homogenized and incubated in 1 ml of saline at 37°C for 20 min, after which 2 ml of acetone with internal standard was added for PG extraction. Homogenates were transferred to screw-top tubes silanized with Sigmacote (Sigma) and centrifuged to remove proteins. Supernatants were washed three times with 2 ml of *n*-hexane to remove non-polar components. The remaining fractions were acidified with 20 μl of 1M formic acid and extracted with 2 ml of chloroform containing 0.01% BHT. Chloroform fractions were concentrated under a stream of N_2_ gas, transferred to silanized microinserts (MicroSolv, 9502S-02ND-RS, Leland, NC), dried again under a stream of N_2_, and redissolved in 20 μl 1:1 methanol:water. Ten μL were injected into the UPLC-MS system for PG analysis. UPLC-MS/MS analysis was performed using a Waters triple quadrupole TQ-S MS (Waters Corporation) in multiple reaction monitoring (MRM) mode with electrospray ionization operated in negative ion mode as previously described (13, 16). PG quantification was performed using PGE_2_-d_9_ as an internal standard.

### Analysis of brain free arachidonic acid

Pulverized brain tissues (∼20 mg) were weighed and sonicated in 90 μl of methanol containing 0.2% BHT and 200 ng of deuterated arachidonic acid (20:4n6-d_8_, Cayman Chemical) as an internal standard. Samples were centrifuged (12,000 *g* 15 min) and a 10 μl aliquot of the supernatant was dried under a stream of N_2_ before being redissolved in 1 ml acetonitrile:2-propanol:water (1:1.28:1.28 by volume). Ten μL were injected into a Waters ACQUITY UPLC system coupled to a quadrupole time-of-flight Waters Synapt XS MS for analysis as we previously described (16, 24, 25).

### Histochemical analysis

In situ unfixed, MW-fixed, and 3 min boiled-fixed whole brains were collected for histochemical analysis. Briefly, following post-fixation in 4% paraformaldehyde and sucrose infiltration for cryopreservation, brains were frozen and then sectioned serially in the coronal plane at 8 μm using a Leica CM3050 S cryostat (Leica Biosystems). Sections were thaw-mounted directly onto Superfrost Plus glass slides (VWR International) and allowed to dry overnight at room temperature. To facilitate morphological assessment, sections were stained with hematoxylin and eosin according to a protocol adapted in our lab for use with fixed, frozen tissue (26).

### Statistical analysis

All statistical and regression analyses were performed using GraphPad Prism 10 (GraphPad). Brain temperature dynamics were analyzed using nonlinear regression analysis. The means between two groups were analyzed using a two-tailed, unpaired Student’s *t* test, and between multiple groups using one-way ANOVA with Tukey’s *post hoc* test. F-test was used to determine differences between standard deviations. Values were considered significant with *P*-value < 0.05 and are expressed as mean ± SD.

## Results

### Increase in brain temperature during boiling

**Fig. 1:**
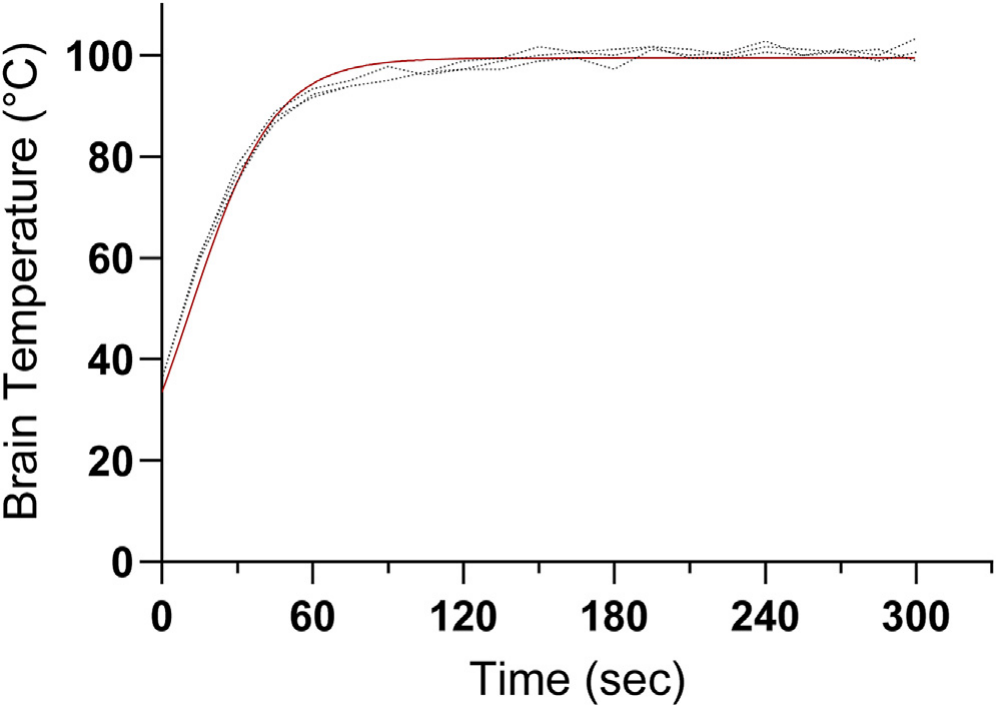
Temperature increase in the mouse brain placed in boiling saline. A silicone-insulated thermocouple was inserted into the cranial space of decapitated mice, fixed in place with surgical thread, and incubated between heating pads until brain internal temperatures were at physiological levels. Whole heads were placed in boiling saline and temperature was recorded every 15 s for 5 min. Placement of the probe was verified during craniotomy and no additional alterations in brain structure were observed. The nonlinear regression curve is shown in red. Data for individual brains are shown with dotted lines. Temperatures at 1.5 and 3 min are 96 and 100°C, respectively. *n (number of animals)* = 3.

To record the temperature dynamics in a mouse brain during boiling, severed heads were probed with an insulated thermocouple as described in the Methods. When placed in boiling saline, temperatures within the brain increased rapidly within the first minute, plateaued near 100°C by 144 s, and maintained this temperature for the remainder of the 5 min observation period (Fig. 1). At 1.5 min of boiling, the internal temperature for mouse brain was 96°C according to the nonlinear regression analysis (n = 3). After 3 min of boiling, the internal brain temperature was 100 ± 1.74°C. Importantly, boiling did not disrupt gross brain morphology and the hippocampus remained intact, while the hippocampus is often lost as an artifact of MW when overheated (supplemental Fig. S1). The advantage of structural preservation potentiates that boiling can be used to fix tissue for regional analysis of brain metabolites.

### PGs are not synthesized postmortem in brains boiled for 3 min

**Fig. 2:**
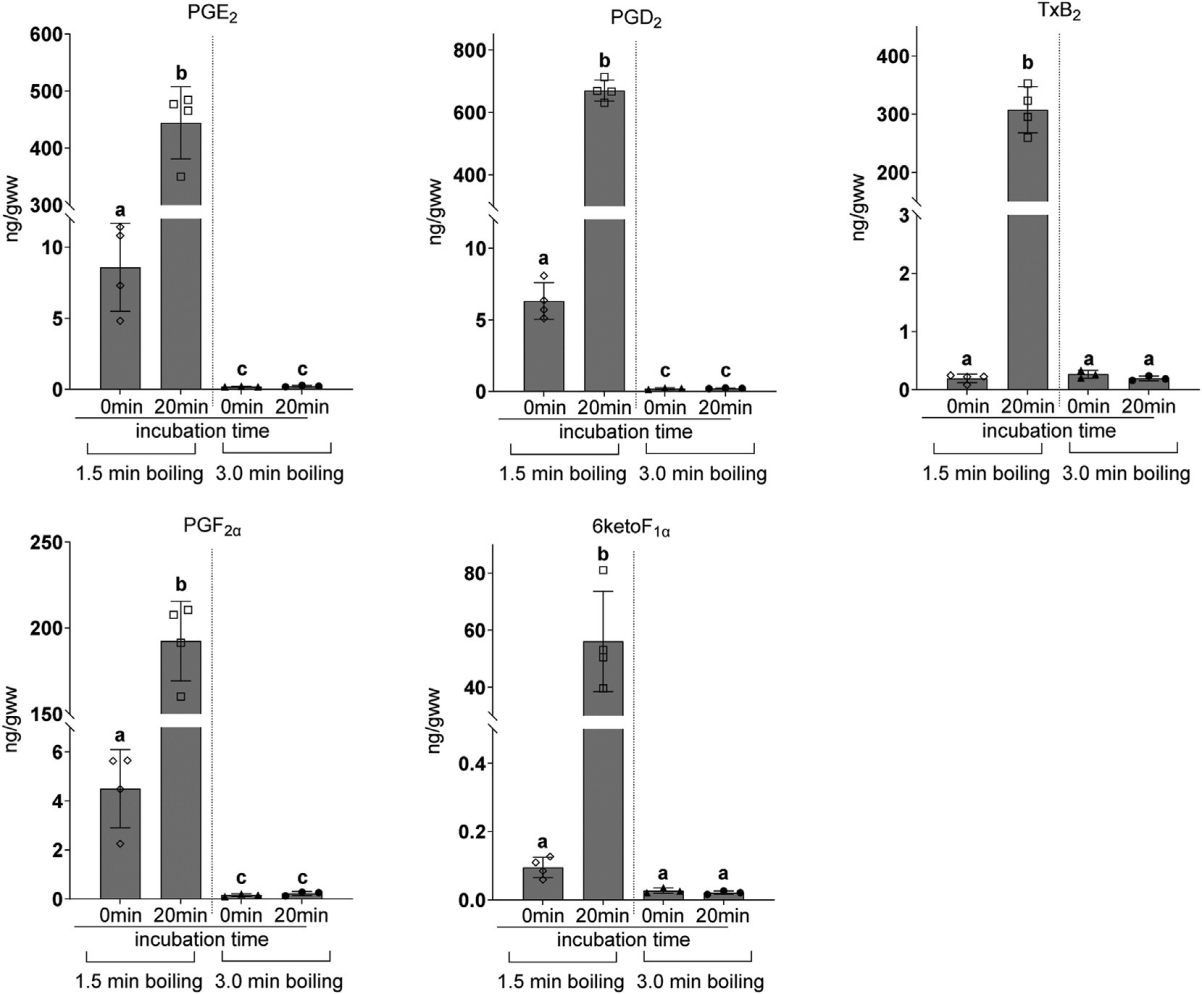
The effect of boiling on brain PG postmortem synthesis, Mouse brains in the intact cranial vault were boiled for 1.5 or 3 min. After pulverization and homogenization, brain samples were analyzed immediately or after incubation at 37°C for 20 min in 1 ml of saline to evaluate remnant prostanoid (PG) synthesis activity. PG were quantified by UPLC-MS against stable isotope labeled internal standard. Values are mean ± standard deviation (n = 3–4) with individual values. Values that do not share the same letter are statistically different (*p* < 0.05, one-way ANOVA with Tukey’s *post hoc* test).

To determine whether boiling was sufficient for preventing postmortem induction of PG, PG levels were quantified from mouse brains boiled for 1.5 and 3 min immediately after tissue collection, and after tissue homogenate incubation at 37°C for 20 min (Fig. 2). In non-incubated brains, PGE_2_, PGD_2_, and 6ketoF_1α_ were significantly higher in 1.5 min boiled brains compared to 3 min boiled samples. Homogenate incubation for 20 min significantly increased all PG levels in 1.5 min boiled samples but had no effect on 3-min boiled samples. These data indicate that 3 min of boiling is sufficient to inactivate brain PG synthesis during tissue handling. In all other experiments using this fixation method, a total boiling time of 3 min was used.

### Sub-boiling temperatures do not fully prevent postmortem PG alterations

**Fig.3:**
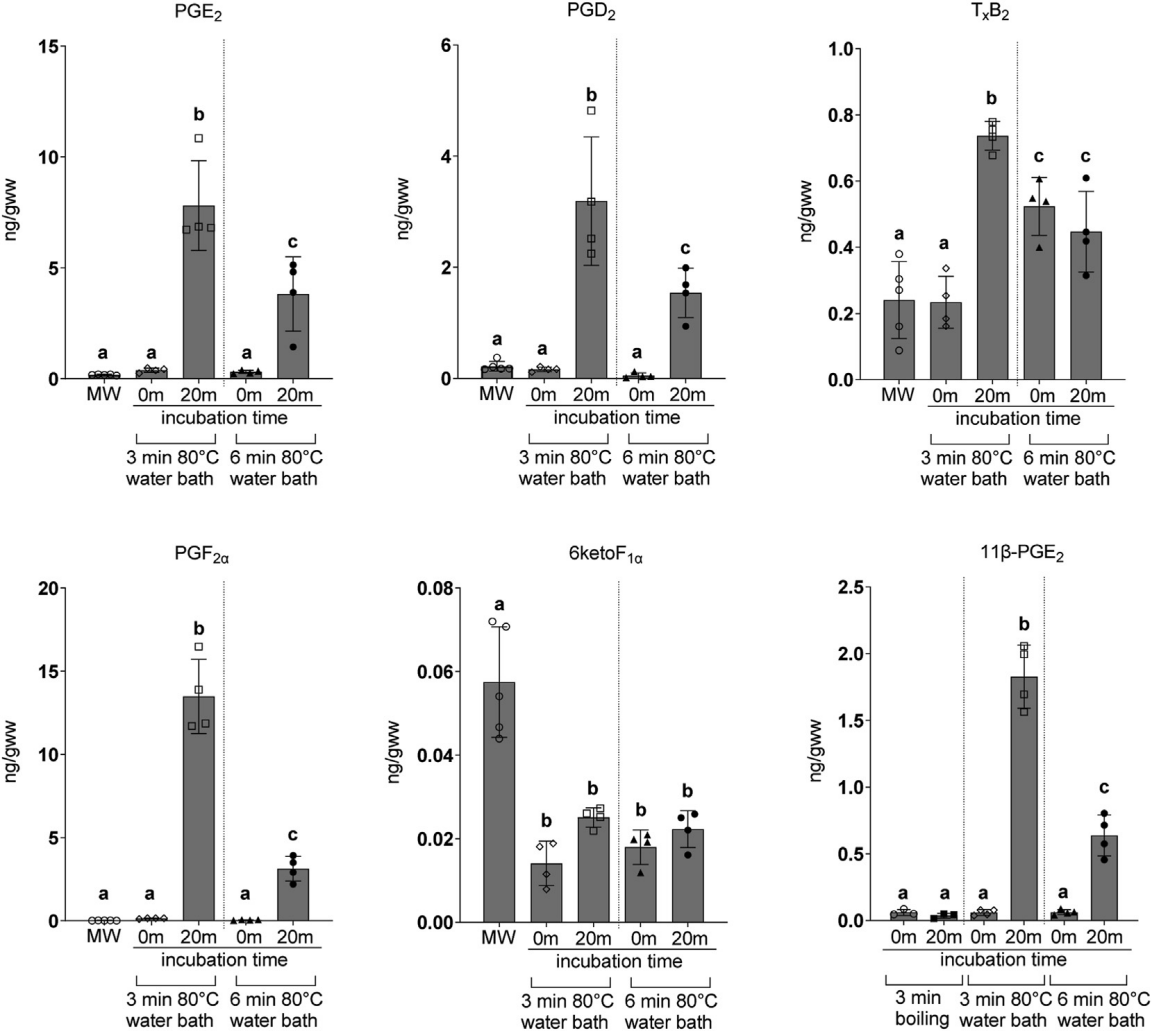
The effect of heating at 80°C on brain PG postmortem synthesis. Mouse brains in the intact cranial vault were submerged in a beaker of saline equilibrated in an 80°C water bath for 3 and 6 min. After pulverization and homogenization, brain samples were analyzed before and after incubation at 37°C for 20 min in 1 ml of saline to evaluate remnant prostanoid (PG) synthesis activity. PG were quantified by UPLC-MS against stable isotope labeled internal standard. Values are mean ± standard deviation (n = 3–4) with individual values. Values that do not share the same letter are statistically different (*p* < 0.05, one-way ANOVA with Tukey’s *post hoc* test).

To further investigate the temperature requirements for enzyme deactivation, mice were decapitated into saline maintained at 80°C, and brains were collected and frozen after 3 or 6 min incubation. PGs were quantified before or after tissue homogenate incubation at 37°C for 20 min to validate the residual enzymatic activity (Fig. 3). In all non-incubated samples, PGE_2_, PGD_2_, and PGF_2α_ were not different from MW brains. However, T_x_B_2_ was increased in the 6 min heated samples, while 6ketoF_1α_, was decreased. Homogenate incubation at 37°C for 20 min significantly increased PGE_2_, PGD_2_, PGF_2α_, and T_x_B_2_ when compared to non-incubated samples. Importantly, PG increase was up to ∼200-fold lower as compared to 1.5 min boiled samples (Fig. 2), indicating lower, if any, COX activity. Exposure to 80°C for 6 min reduced PG levels during incubation as compared to 3 min, but did not prevent PG increase (Fig. 3). To validate whether increased PGs are derived from an esterified pool of nonenzymatically produced isoprostanes (isoPG) by lipases still active after heating at 80°C, we measured levels of one of the most abundant isoPG, 11β-PGE_2_ (12, 13). In samples analyzed without incubation, levels of 11β-PGE_2_ were not different between tissues from 3 min boiled brains, or those fixed at 80°C for 3 and 6 min. However, 20 min homogenate incubation increased 11β-PGE_2_ levels in brains fixed at 80°C for 3 or 6 min, but not in 3 min boiled brains. Importantly, the dynamics and magnitude of 11β-PGE_2_ increase during incubation were similar to other PG, suggesting that measured PG might originate from non-enzymatically oxidized esterified 20:4n6 released by the residual lipase activity in the samples exposed to 80°C.

These data indicate that while COX activity is reduced in brains submerged in 80°C saline for 3 or 6 min, there is still a postmortem increase in PG levels after 20 min homogenate incubation, likely due to the release of an esterified isoPG pool.

Additionally, we found a decrease in 6ketoF_1α_ levels for all brains submerged in 80°C saline when compared to MW brains, indicating that degradative pathways are still active for some PG. It is well established that PGI_2_ is unstable and is rapidly, nonenzymatically converted to 6ketoF_1α_ with a half-life of 2 min in some aqueous solutions, with a complete loss of biological activity in 20 min (27, 28). However, enzymatic conversion of PGI_2_ to 6,15diketoPGF_1α_ (28) might decrease PGI_2_ availability for degradation to 6ketoF1α if the enzymes are not completely deactivated under lower fixation temperatures, attributing an additional artifact to the PG analysis.

Together, these data support the advantage of 3 min boiling fixation over lower temperatures with a longer duration of submersion in saline.

### Boiling does not arrest postmortem free arachidonic acid increase in the brain

**Figure 4:**
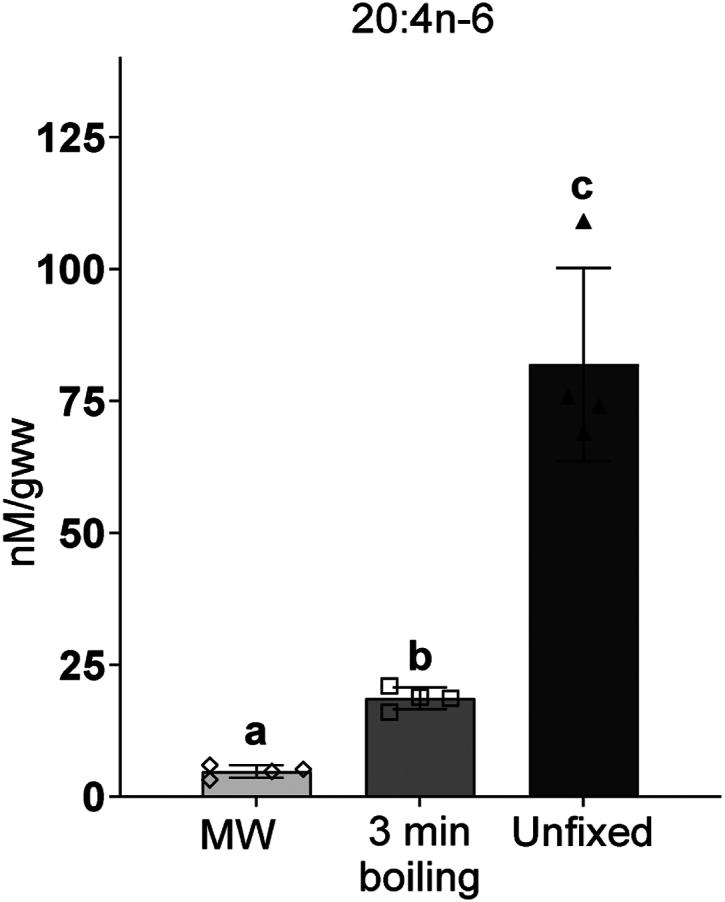
The effect of boiling on brain-free arachidonic acid release. Free arachidonic acid (20:4n-6) from microwave irradiated (MW), 3 min boiled, and unfixed mouse brain tissues were analyzed by UPLC-MS against stable isotope labeled internal standard. Values are mean ± standard deviation (n = 4) with individual values. Values that do not share the same letter are statistically different (*p* < 0.05, one-way ANOVA with Tukey’s *post hoc* test).

It is well documented that 20:4n-6 is dramatically increased immediately in the brain postmortem (25, 29, 30) as a result of immediate PLA_2_ activation after tissue energy depletion. To test if boiling prevents 20:4n-6 release, free 20:4n-6 levels were quantified in MW, boiled, and unfixed brain tissue. Compared to tissue samples fixed by MW, both boiled and unfixed tissue had significant 4-fold and 17-fold increased levels of 20:4n-6, respectively (Fig. 4). These data suggest that boiling is not sufficient to rapidly deactivate phospholipase activity in the ischemic brain and confirms our previous speculation that O_2_ availability but not 20:4n-6 release regulates PG production under brain ischemia (16).

### Basal and LPS-induced PG levels are not different in boiled and MW brains

**Fig. 5:**
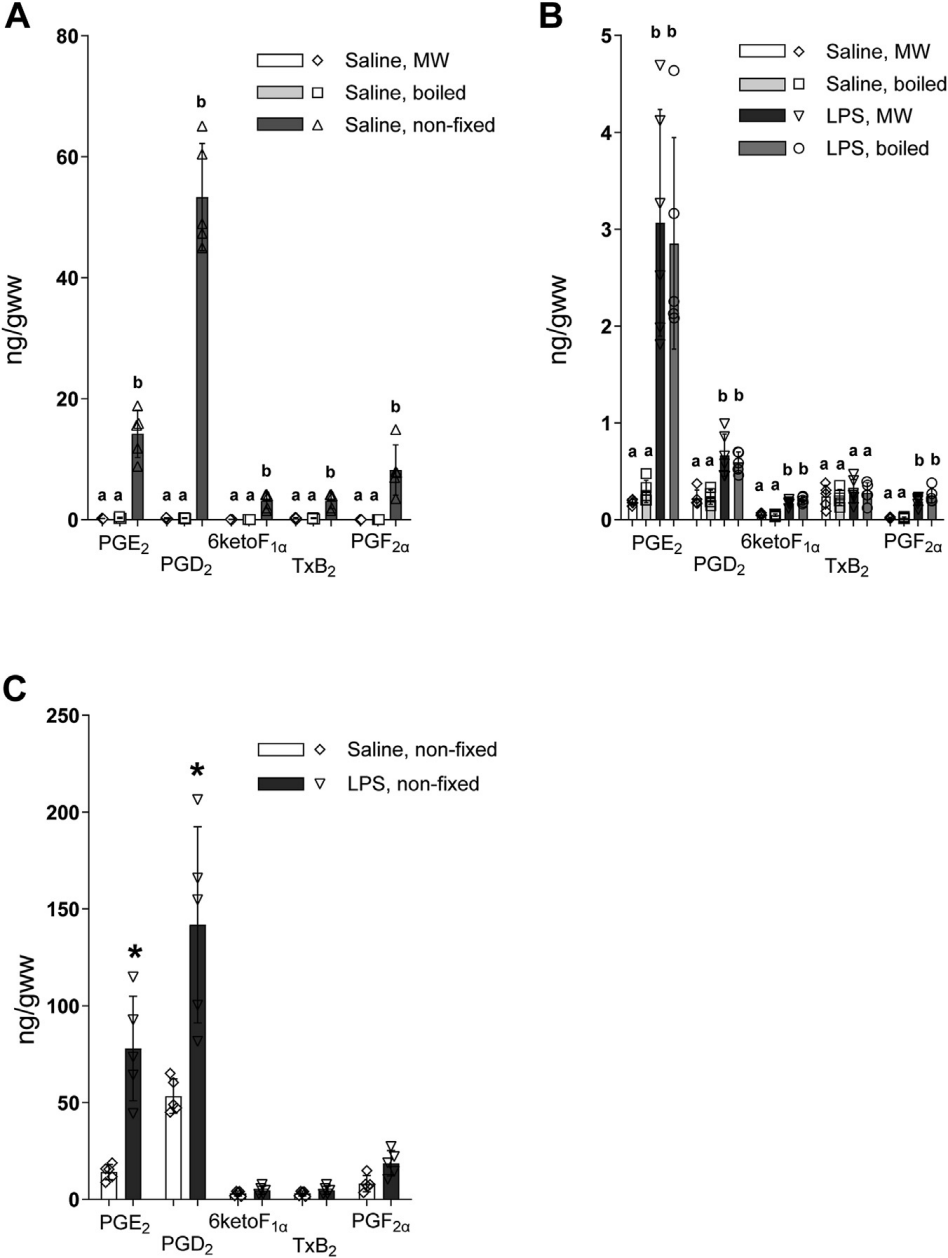
The efficacy of boiling and MW fixation in preventing brain postmortem PG alterations under basal conditions and upon LPS treatment. Mice were treated with lipopolysaccharide (LPS) in saline (i.p., 1 mg/kg) or saline vehicle alone. After 3 h, brains were collected from unfixed, microwave irradiated (MW), and boiled mouse brains and subject to analysis by UPLC-MS/MS. Values are mean ± standard deviation (n = 5–6) with individual values. A: Average values for prostanoids (PG) in unfixed, MW, and boiled brain tissue from saline treated mice. For each PG, values that do not share the same letter are statistically different (*p* < 0.05, one-way ANOVA with Tukey’s *post hoc* test, performed separately for each PG). B: Average values for PG in MW and boiled brain tissue from saline and LPS-treated mice. For each PG, values that do not share the same letter are statistically different (*p* < 0.05, one-way ANOVA with Tukey’s *post hoc* test performed separately for each PG). C: Average values for PG in unfixed brain tissue from saline and LPS-treated mice. Values with an asterisk are statistically different (*p* < 0.05, *t* test).

To compare the efficacy of fixation methods for PG quantification under basal conditions and upon stimulation, we quantified basal and LPS-induced PG in unfixed, MW, and boiled mouse brain tissues. Consistent with previous studies (5, 6, 11, 16, 31), unfixed tissue had a significant increase in PGE_2_ (60-fold), PGD_2_ (235-fold), 6ketoF_1α_ (60-fold), T_X_B_2_ (13-fold), and PGF_2α_ (420-fold) analyzed at basal conditions, with the highest increase in PGD_2_ (Fig. 5A). Both MW and boiling prevented postmortem PG increase with equal efficacy, as basal PG levels were not different between the two groups (Fig. 5A, B). Similarly, there were no differences between MW and boiled samples in all PG analyzed after LPS treatment (Fig. 5B). All PG, except for T_X_B_2_, were significantly increased compared to saline-treated animals when measured after MW or boiling (Fig. 5B). Specifically, LPS increased PGE_2_ (13- and 12-fold), PGD_2_ (3- and 3-fold), 6ketoF_1α_ (3- and 4-fold), and PGF_2α_ (10- and 13-fold) in MW and boiled tissues, respectively, compared to basal levels. In unfixed tissue, we did not detect significant changes in 6ketoF_1α_ or PGF_2α_ after LPS treatment, while a lesser difference was detected for PGE_2_ (5-fold) (Fig. 5C).

These data indicate that boiling and MW are similarly effective in preventing postmortem PG alterations and recapitulate the importance of adequate tissue fixation for analysis of true PG changes under different conditions. Importantly, the relative standard deviation values for PG were not different between MW and boiled groups as analyzed with F-test, indicating equal variability between the two fixation methods.

## Discussion

It is well documented that the deactivation of enzymes for PG synthesis is needed for the quantification of accurate *in situ* PG levels in different tissues, including the brain (5, 6, 7, 11). Rapid enzyme deactivation was considered essential for PG analysis because PG synthesis was assumed to be activated immediately upon ischemia onset. Thus, enzyme heat denaturation with MW is currently the gold standard for tissue fixation before PG quantification, providing a rapid (1–2 s) tissue heating to 80–90°C in anesthetized animals (10).

However, we have recently reported that free 20:4n-6 is significantly increased during global brain ischemia but is not converted into PG until metabolically active tissue is exposed to atmospheric O_2_ during craniotomy (16). In this study, we demonstrated that in the ischemic brain, O_2_ is rapidly depleted preceding the increase in 20:4n-6 levels needed for PG production (16); thus, PG synthesis does not occur in ischemic tissue. Based on this proposed mechanism, we hypothesized that slow heat denaturation by boiling in saline would be sufficient to prevent PG synthesis if enzymes are inactivated before craniotomy, providing an alternative to the MW tissue fixation method for PG quantification.

In the present study, we validated this alternative method for tissue fixation by boiling. We demonstrated that despite an increase in 20:4n-6 levels after 3 min of boiling (Fig. 4), there was ultimately no difference in basal PG levels between brains fixed slowly by boiling and those fixed rapidly with MW (Fig. 5B). The results of our brain incubation experiments indicate that boiling fixation denatures enzymes necessary for PG synthesis within the cranial vault before tissue exposure to O_2_, preventing postmortem increase in PG during craniotomy and tissue processing (Fig. 2). Of note, in compliance with the University of North Dakota IACUC protocol, which requires anesthetization prior to these forms of euthanasia, we used isoflurane prior to microwave fixation or decapitation into boiling saline. Because all control and experimental animal groups used for PG analysis were subjected to the same anesthesia conditions, it is unlikely that anesthesia contributed to the observed efficiency of the boiling procedure tested in the study.

We also investigated lower temperature at longer duration fixation in saline and we found that while PG increase was prevented when analyzed without further incubation, 3 and 6 min in 80°C saline was not sufficient to prevent postmortem PG increase during incubation at 37°C (Fig. 3). Notably, the magnitude of PG increase under these conditions was up to ∼200-fold lower compared to under-fixed tissue and correlated with the dynamics of 11β-PGE_2_ increase (Figs. 2 and 3). These data suggest that measured PG might originate from non-enzymatically oxidized esterified 20:4n6 released by residual lipase activity in the samples exposed to 80°C. The presence of these releasable esterified isoPG pools was previously described for the brain (12, 13). We also found that attempted fixation at this temperature permits degradation of some PG as was shown for 6ketoPGF_1α_, a stable degradation product of PGI_2_ (Fig. 3). In this mechanism, enzymatic conversion of PGI_2_ to 6,15diketoPGF_1α_ (28) might decrease PGI_2_ availability for non-enzymatic degradation to 6ketoF1α (27, 28) if the enzymes are not completely deactivated under lower fixation temperatures, attributing an additional artifact to the PG analysis. For these reasons, we concluded that boiling fixation was advantageous over fixation in saline at a lower temperature.

We propose that boiling fixation may be a useful method for preventing postmortem alterations in other oxygen-dependent metabolites, such as those produced by lipoxygenase and cytochrome P450 enzymes if a rapid release from releasable pre-esterified pools does not contribute to regulation of their levels under ischemia. Further, these results substantiate the equal efficacy of MW and boiling fixation prior to PG quantification after pro-inflammatory stimulation with LPS (Fig. 5B). Importantly, our analysis revealed no difference in standard deviation for all PG analyzed either at baseline or following LPS administration, suggesting similar variability in these fixation methods.

It is reasonable to consider that heating by boiling or MW may lead to the loss of some metabolites before extraction. Some energy metabolites, namely nucleoside phosphates, are susceptible to degradation during exposure to 10 kW MW, with brain temperatures reaching 85°C (32). However, PG are not as heat-labile as other compounds, as demonstrated in our previous study (7), in which we did not observe significant degradation of a PG mixture incubated at MW temperatures. Similar results were obtained with exogenous stable isotope-labeled PG injected into brain before MW (6). Given that the boiling method showed no statistical difference in PG mass for all treatments when compared to MW (Fig. 54B), any loss of PG is likely within this same range.

Although both MW and boiling provide comparable results in preventing postmortem PG synthesis, our new boiling fixation method has substantial advantages. In contrast to MW fixed brains, no gross tissue loss or morphological alteration was observed in boiled brains, permitting regional metabolite analysis (supplementary Fig. S1). Additionally, boiling requires only basic supplies and materials already present in most laboratories, whereas MW fixation necessitates specialized equipment that is costly and not widely available. We expect that animals with larger brains than the typical adult mouse may require longer boiling periods to denature enzymes in deep regions of the brain, while smaller animals, such as neonatal mice, may not require the full 3 min of boiling, and the method will require boiling time adaptation for the animal size used for analysis.

In summary, we demonstrate that decapitation into boiling saline is a reliable alternative method for preventing postmortem induction of PG to facilitate the assessment of true endogenous levels in the mouse brain. This method is similar to MW in efficacy and PG quantification variability but possesses compelling advantages in terms of reproducibility in tissue structure preservation and fixation, expense, and accessibility to most laboratories. Additional study is required to determine its application for other labile metabolites.

## Data availability

All data are contained in this manuscript.

## Supplemental data

This article contains supplemental data.

## Conflict of interest

The authors declare that they have no conflicts of interest with the contents of this article.

## References

[1] Kellawan J.M., Peltonen G.L., Harrell J.W., Roldan-Alzate A., Wieben O., Schrage W.G. (2020). Differential contribution of cyclooxygenase to basal cerebral blood flow and hypoxic cerebral vasodilation. Am. J. Physiol. Regul. Integr. Comp. Physiol..

[2] Rand A.A., Barnych B., Morisseau C., Cajka T., Lee K.S.S., Panigrahy D. (2017). Cyclooxygenase-derived proangiogenic metabolites of epoxyeicosatrienoic acids. Proc. Natl. Acad. Sci. U. S. A..

[3] Seeger D.R., Golovko S.A., Grove B.D., Golovko M.Y. (2021). Cyclooxygenase inhibition attenuates brain angiogenesis and independently decreases mouse survival under hypoxia. J. Neurochem..

[4] Vane J.R., Bakhle Y.S., Botting R.M. (1998). Cyclooxygenases 1 and 2. Annu. Rev. Pharmacol. Toxicol..

[5] Anton R.F., Wallis C., Randall C.L. (1983). In vivo regional levels of PGE and thromboxane in mouse brain: effect of decapitation, focused microwave fixation, and indomethacin. Prostaglandins.

[6] Farias S.E., Basselin M., Chang L., Heidenreich K.A., Rapoport S.I., Murphy R.C. (2008). Formation of eicosanoids, E2/D2 isoprostanes, and docosanoids following decapitation-induced ischemia, measured in high-energy-microwaved rat brain. J. Lipid Res..

[7] Golovko M.Y., Murphy E.J. (2008). An improved LC-MS/MS procedure for brain prostanoid analysis using brain fixation with head-focused microwave irradiation and liquid-liquid extraction. J. Lipid Res..

[8] Crockard H.A., Bhakoo K.K., Lascelles P.T. (1982). Regional prostaglandin levels in cerebral ischemia. J. Neurochem..

[9] Petroni A., Bertazzo A., Sarti S., Galli C. (1989). Accumulation of arachidonic acid cyclo- and lipoxygenase products in rat brain during ischemia and reperfusion: effects of treatment with GM1-lactone. J. Neurochem..

[10] Murphy E.J. (2010). Brain fixation for analysis of brain lipid-mediators of signal transduction and brain eicosanoids requires head-focused microwave irradiation: an historical perspective. Prostaglandins Other Lipid Mediat..

[11] Brose S.A., Golovko M.Y. (2013). Eicosanoid post-mortem induction in kidney tissue is prevented by microwave irradiation. Prostaglandins, Leukot. Essent. Fatty Acids.

[12] Brose S., Baker A., Golovko M. (2013). A fast one-step extraction and UPLC–MS/MS analysis for E2/D2 series prostaglandins and isoprostanes. Lipids.

[13] Brose S.A., Thuen B.T., Golovko M.Y. (2011). LC/MS/MS method for analysis of E2 series prostaglandins and isoprostanes. J. Lipid Res..

[14] Golovko M.Y., Faergeman N.J., Cole N.B., Castagnet P.I., Nussbaum R.L., Murphy E.J. (2005). α-Synuclein gene deletion decreases brain palmitate uptake and alters the palmitate metabolism in the absence of α-synuclein palmitate binding. Biochemistry.

[15] Seeger D.R., Golovko S.A., Golovko M.Y. (2020). Blood–brain barrier is the major site for a rapid and dramatic prostanoid increase upon brain global ischemia. Lipids.

[16] Seeger D.R., Schofield B., Besch D., Golovko S.A., Kotha P., Parmer M. (2023). Exogenous oxygen is required for prostanoid induction under brain ischemia as evidence for a novel regulatory mechanism. J. Lipid Res..

[17] Kalmbach A.S., Waters J. (2012). Brain surface temperature under a craniotomy. J. Neurophysiol..

[18] Cao C., Matsumura K., Ozaki M., Watanabe Y. (1999). Lipopolysaccharide injected into the cerebral ventricle evokes fever through induction of cyclooxygenase-2 in brain endothelial cells. J. Neurosci..

[19] Cao C., Matsumura K., Yamagata K., Watanabe Y. (1995). Induction by lipopolysaccharide of cyclooxygenase-2 mRNA in rat brain; its possible role in the febrile response. Brain Res..

[20] Catorce M.N., Gevorkian G. (2016). LPS-Induced murine neuroinflammation model: main features and suitability for pre-clinical assessment of nutraceuticals. Curr. Neuropharmacol..

[21] de Vries H.E., Hoogendoorn K.H., van Dijk J., Zijlstra F.J., van Dam A.M., Breimer D.D. (1995). Eicosanoid production by rat cerebral endothelial cells: stimulation by lipopolysaccharide, interleukin-1 and interleukin-6. J. Neuroimmunol..

[22] Jangula A., Murphy E.J. (2013). Lipopolysaccharide-induced blood brain barrier permeability is enhanced by alpha-synuclein expression. Neurosci. Lett..

[23] Qin L., Wu X., Block M.L., Liu Y., Breese G.R., Hong J.-S. (2007). Systemic LPS causes chronic neuroinflammation and progressive neurodegeneration. Glia.

[24] Brose S.A., Golovko S.A., Golovko M.Y. (2016). Fatty acid biosynthesis inhibition increases reduction potential in neuronal cells under hypoxia. Front. Neurosci..

[25] Golovko S.A., Golovko M.Y. (2018). Plasma unesterified fatty-acid profile is dramatically and acutely changed under ischemic stroke in the mouse model. Lipids.

[26] Carson F.L., Hladik C., Cappellano C.H. (2015).

[27] Lewis P.J., Dollery C.T. (1983). Clinical pharmacology and potential of prostacyclin. Br. Med. Bull..

[28] Wong P.Y., Sun F.F., McGiff J.C. (1978). Metabolism of prostacyclin in blood vessels. J. Biol. Chem..

[29] Bazan N.G. (1970). Effects of ischemia and electroconvulsive shock on free fatty acid pool in the brain. Biochim. Biophys. Acta.

[30] Bazan N.G. (1971). Changes in free fatty acids of brain by drug-induced convulsions, electroshock and anesthesia. J. Neurochem..

[31] Kratz D., Wilken-Schmitz A., Sens A., Hahnefeld L., Scholich K., Geisslinger G. (2022). Post-mortem changes of prostanoid concentrations in tissues of mice: impact of fast cervical dislocation and dissection delay. Prostaglandins Other Lipid Mediat..

[32] Srivastava S., Kashiwaya Y., Chen X., Geiger J.D., Pawlosky R., Veech R.L. (2012). Microwave irradiation decreases ATP, increases free [Mg2+], and alters in vivo intracellular reactions in rat brain. J. Neurochem..

