# Supplementary material for: A simplified method for preventing postmortem alterations of brain prostanoids for true in situ level quantification
Source: J Lipid Res. 2024 Jun 21;65(7):100583. doi: 10.1016/j.jlr.2024.100583 (PMC11301166; doi:10.1016/j.jlr.2024.100583)
Supplement: Supplementary Figures [file mmc1.pdf]

Supplementary Figure S1. ***In situ*** **boiling does not disrupt gross brain morphology**

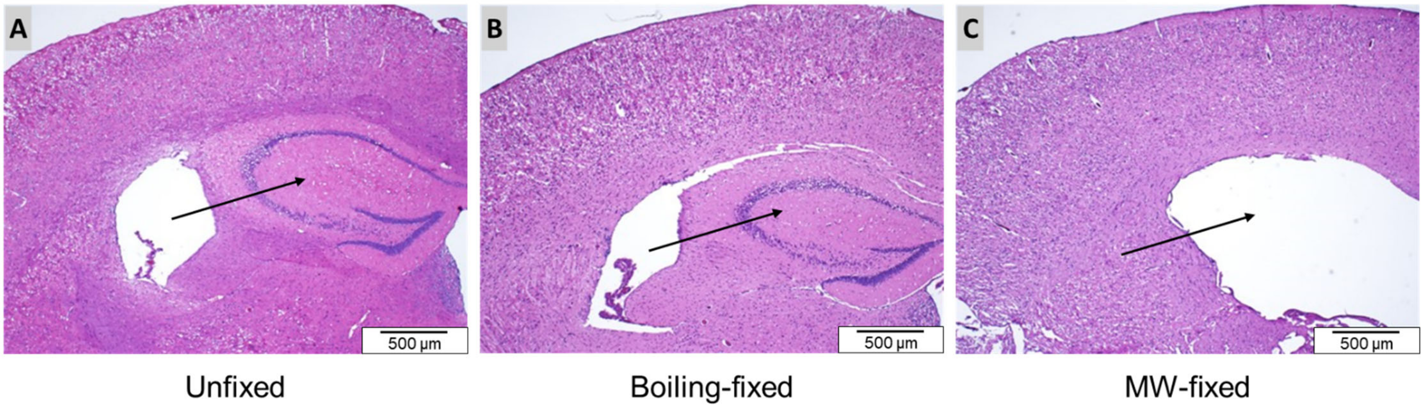

In situ unfixed (**A**), *in situ* 3 min boiling-fixed (**B**), and *in situ* microwave irradiation-fixed (MW, **C**) whole brains were collected for histochemical analysis and immersion fixed in 4% paraformaldehyde overnight followed by overnight cryopreservation in 15% sucrose and overnight cryopreservation again in 30% sucrose. Brains were frozen on dry ice and coronally sliced at 8 µm using a Leica CM 3050S cryostat. Slices were stained with hematoxylin and eosin and imaged at 4x using brightfield on an Olympus BX53 light microscope. Brain structures, including hippocampus (black arrow), are preserved in boiled brain (**B**), but often lost or obscured in MW brain (**C**).
